# Negative Effects of Latent Toxoplasmosis on Mental Health

**DOI:** 10.3389/fpsyt.2019.01012

**Published:** 2020-02-18

**Authors:** Jaroslav Flegr, Jiří Horáček

**Affiliations:** ^1^Division of Biology, Faculty of Science, Charles University in Prague, Prague, Czechia; ^2^Applied Neurosciences and Brain Imagination, National Institute of Mental Health, Klecany, Czechia

**Keywords:** psychiatric diseases, mental health, prevalence, risk factors, etiology, *Toxoplasma*, infection hypothesis

## Abstract

Infection by the parasite *Toxoplasma*, which affects about 33% of world population, is associated with an increased risk of several mental health disorders, the most strongly with schizophrenia. It is unknown whether schizophrenia is associated with this infection the most strongly, or whether this association has just been the most intensively studied for historical reasons. We used the data from 6,367 subjects tested for toxoplasmosis who took part in an internet survey to search for associations of these infections with 24 mental health disorders and evidence of otherwise impaired mental health. The typical symptom associated with toxoplasmosis was anxiety, and the typical toxoplasmosis-associated disorders were autism (OR = 4.78), schizophrenia (OR = 3.33), attention deficit hyperactivity disorder (OR = 2.50), obsessive compulsive disorder (OR = 1.86), antisocial personality disorder (OR = 1.63), learning disabilities (OR = 1.59), and anxiety disorder (OR = 1.48). Toxoplasmosis could play a substantial role in the etiopathogenesis of mental health disorders and its association with schizophrenia is the second strongest association, after autism.

## Introduction

About one third of the world population is infected with the coccidian parasite *Toxoplasma gondii*. The course of postnatally acquired toxoplasmosis in immunocompetent subjects is mild, and therefore so-called latent toxoplasmosis has been mostly considered as clinically insignificant. However, the results of recent studies show that this picture could be wrong. Specifically, the *Toxoplasma*-seropositivity has been associated with the increased risk of many mental and physical health disorders (1) and between-country differences in seroprevalence of toxoplasmosis could explain 23% of the total variability in disease burden in European countries (2). The connection between certain mental health disorders, especially schizophrenia, and toxoplasmosis has been documented beyond any reasonable doubt, for review see (3, 4). Moreover, the causal role of toxoplasmosis in the development of schizophrenia has been confirmed by a longitudinal study (5). It has even been documented that the changes in brain morphology that are characteristic of schizophrenia, such as gray matter reduction in frontal and temporal cortices, caudate, median cingulate, and thalamus, are in fact typical for *Toxoplasma*-seropositive schizophrenia patients (6). Congruently, the *Toxoplasma*-seropositive patients express more prominent positive symptoms of schizophrenia (7, 8) and have a 15-times higher probability of having a continuous course of disease than the *Toxoplasma*-free patients (9). Far fewer studies have shown the association of toxoplasmosis with other mental health disorders. About 10 studies have shown the association of toxoplasmosis with bipolar disorder, and less than five with obsessive compulsive disorder, learning disorder, autism, and anxiety disorder; for reviews see (4, 10).

It is not known whether the association of *Toxoplasma* with schizophrenia is the strongest or whether it is just most often studied. To address this question, we performed a systematic search for any association between *Toxoplasma*-seropositivity and 22 common mental health disorders. However, the prevalence of certain disorders, e.g., schizophrenia, is relatively low and doing many statistical tests requires performing rigorous correction for multiple tests. For these reasons, we needed to analyze a very large data set. To this end, we utilized data from a recently obtained, large internet-based cohort study performed on about 60,000 members of the general internet population.

## Materials and Methods

### Study Population

The data was originally collected for the purpose of another study (11). The subjects were invited to participate in the study using a Facebook-based snowball method (12) by advertisements published in various papers and electronic media, as well as TV and radio broadcasting. The invitation to participate in a “study testing certain evolutionary psychological and parasitological hypotheses, containing many questions related to sexual life” was also posted on the wall of the Facebook page “Guinea pigs” (“Pokusní králíci” in Czech) for Czech and Slovak nationals willing to take part in diverse ethological and psychological projects (www.facebook.com/pokusnikralici). The participants were informed about the aims of the study on the first page of the electronic questionnaire. They were also provided with the following information: “The questionnaire is anonymous and the obtained data will be used exclusively for scientific purposes. Your cooperation in the project is voluntary and you can terminate it at any time by closing this web page. You can also skip any uncomfortable questions; however, the most valuable data is complete data. Only subjects above 15 years old are allowed to take the questionnaire. If you agree to participate in the research and are above 15, press the “Next” button”. The questionnaire was written in Czech language, therefore only Czech and Slovak took part in the study. More information about the composition of the population under study shows the first paragraph of the section Results. Some pages of the questionnaire contained the Facebook share button. These buttons were pressed by 1,660 participants, which resulted in obtaining data from about 59,000 respondents in total between 22^nd^ January 2015 and 24^th^ July 2019. The project, including the method of obtaining an electronic consent to participate in the study, was approved by the Ethical Committee of the Faculty of Science, Charles University (No. 2015/01). The preliminary results obtained by the analysis of approximately one half of current data set, namely data from 3,440 respondents, were already presented in the form of the Letter to editor (13).

### Questionnaires

The electronic survey consisted of 5 already published questionnaires studying various facets of human sexuality (11). The survey also contained an anamnestic questionnaire collecting various socioeconomic, demographic, health related, epidemiologic, and psychological data and three projective psychological tests. Altogether, the survey consisted of more than 700 questions and the mean time necessary to complete it was about 110 min (the mode was 97 min). In the present study, we used only the information about *sex*, *age*, size of place of living (*urbanization*: 6-points ordinal scale – 0: less than 1,000 inhabitants, 1: 1–5 thousand inhabitants, 2: 5–50 thousand inhabitants, 3: 50–100 thousand inhabitants, 4: 100–500 thousand inhabitants, 5: more than 500 thousand inhabitants), mental health-related variables, and *Toxoplasma* infection. The respondents were asked how they rate their physical health status in comparison to other people of the same age (*subjective physical health —* analog scale 0–100, anchored with 0: definitively worse status, 100: definitively better status), how many *drugs prescribed* by doctors they currently take per day, how many of different preparations or *drugs non-prescribed* by doctors they currently take per day (“how many different herbs, food supplements, multivitamins, super-foods etc. do you currently take in per day”), how many times they have used *antibiotics* during past 365 days, how many times they have visited their *primary care doctor* in past 365 days (“not for prevention”), and how many different *medical specialists* they have visited (not for prevention) in the past 5 years. The *coefficient of physical health* was computed as mean Z-score from the answers to six previous questions. The subjects were also asked about their mental health status in comparison to other people of the same age (*subjective mental health –* analog scale 0–100, anchored with 0: definitively worse status, 100: definitively better status), the intensity of any psychiatric *disorders diagnosed by doctors* and *disorders undiagnosed by doctors* using two analog 0–100 scales. Then they were asked to check which mental health diseases they suffer from (both diagnosed and undiagnosed by a clinician) on the list of 24 disorders, see **Table 3**. The list contained also Alzheimer's disease and Parkinson's disease, however, only one subject tested for *Toxoplasma* reported to suffer from Alzheimer's disease and none reported to suffer from Parkinson's disease, therefore the associations between the infections and these two disorders were not analyzed. The disorders reported by each respondent were manually checked and corrected (e.g., manio-depressive disorder/psychosis, cyclophrenia, anxiety-depressive disorder were re-classified from the category “other disorder” into proper categories); and based on this data, the new variable *number of psychiatric diseases* was computed. They were also asked to rate the intensity of suffering from particular neuropsychiatric symptoms (*depression*, *mania*, *phobia*, *anxiety*, and *obsessions*) with moving sliders on analog scales 0–100, anchored with “Never” (0) and “Intensively or frequently” (100). The *coefficient of mental health* was computed as mean Z-score from the answers to eight previous questions. The subjects have been also asked whether they are *Toxoplasma*-infected. They were reminded that *Toxoplasma* is ‘‘a parasite of cats, dangerous especially to pregnant women”. The response ‘‘I do not know, I am not sure'' was set as a default answer which the respondents could change by selecting either ‘‘No, I was tested by a doctor and the result of my laboratory tests was negative'' or ‘‘Yes, I was tested by a doctor and I had antibodies against *Toxoplasma*''. In a similar more recent internet questionnaire study, we asked the participants for the reasons of their testing for toxoplasmosis. About 50% of men and 20% of women were tested for various health-related reasons (probably not for the mental health-related reasons because, at the present time, psychiatrists practically never send their patients for toxoplasmosis tests), 37% of women were tested in relation to their pregnancy, and 40% men and 34% of women were tested in our lab during participation in various research projects during past 20 years. The respondents had three options: they could complete the questionnaire anonymously, they could sign the finished questionnaire with a code obtained after the anonymous registration, or they could sign the finished questionnaire with a code obtained after the non-anonymous registration.

### Statistical Methods

Before statistical analysis, less than 1% of suspicious data (too tall or too short height, too low or too high body mass or age, too short duration of the test, etc.) were filtered out. Several subjects were also excluded because they checked nearly all mental health disorders, including Parkinson's and Alzheimer's disease. The final raw data set containing the data of 6,367 subjects for which the information about toxoplasmosis status and at least some information about mental health is available at figshare: https://doi.org/10.6084/m9.figshare.9335081.v1.

Statistica v.10.0. was used for exploration of data and R v. 3.3.1 for the confirmatory statistical tests. Differences in the prevalence of individual disorders between the *Toxoplasma*-infected and *Toxoplasma*-free subjects have been analyzed with logistic regression with sex, age, urbanization, and *Toxoplasma* seropositivity as the predictors. Both ANCOVA and non-parametric tests (partial Kendall correlation test, R package ppcor) with age, urbanization, and in some analyses also sex as covariates were used for the analysis of ordinal and continuous variables, however, the parametric and more robust but less sensitive non-parametric tests provided qualitatively equivalent results. False discovery rate (preset to 0.20) was controlled with the Benjamini-Hochberg procedure (14).

Terminological note: By the term “prevalence” we mean prevalence of the subjects reporting particular disorder in our data set, not the prevalence or incidence of a particular disorder in the internet or even general population.

## Results

The final data set contained information on the mental health of 16,740 women (mean age 30.9, SD = 11.4) and 20,032 men (mean age 35.5, SD = 12.5); the difference in age between both sexes was significant (t_36770_ = -36.4, p < 0.0001). Among these subjects, 3,698 women (18.7% seropositive) and 2,669 men (9.9% seropositive) provided the information about their toxoplasmosis status. The difference in age between *Toxoplasma*-infected and *Toxoplasma*-free subjects was significant for women (33.2 vs 35.2, p = 0.00003) but non-significant for men (37.6 vs 36.6, p = 0.220).

**Figure 1:**
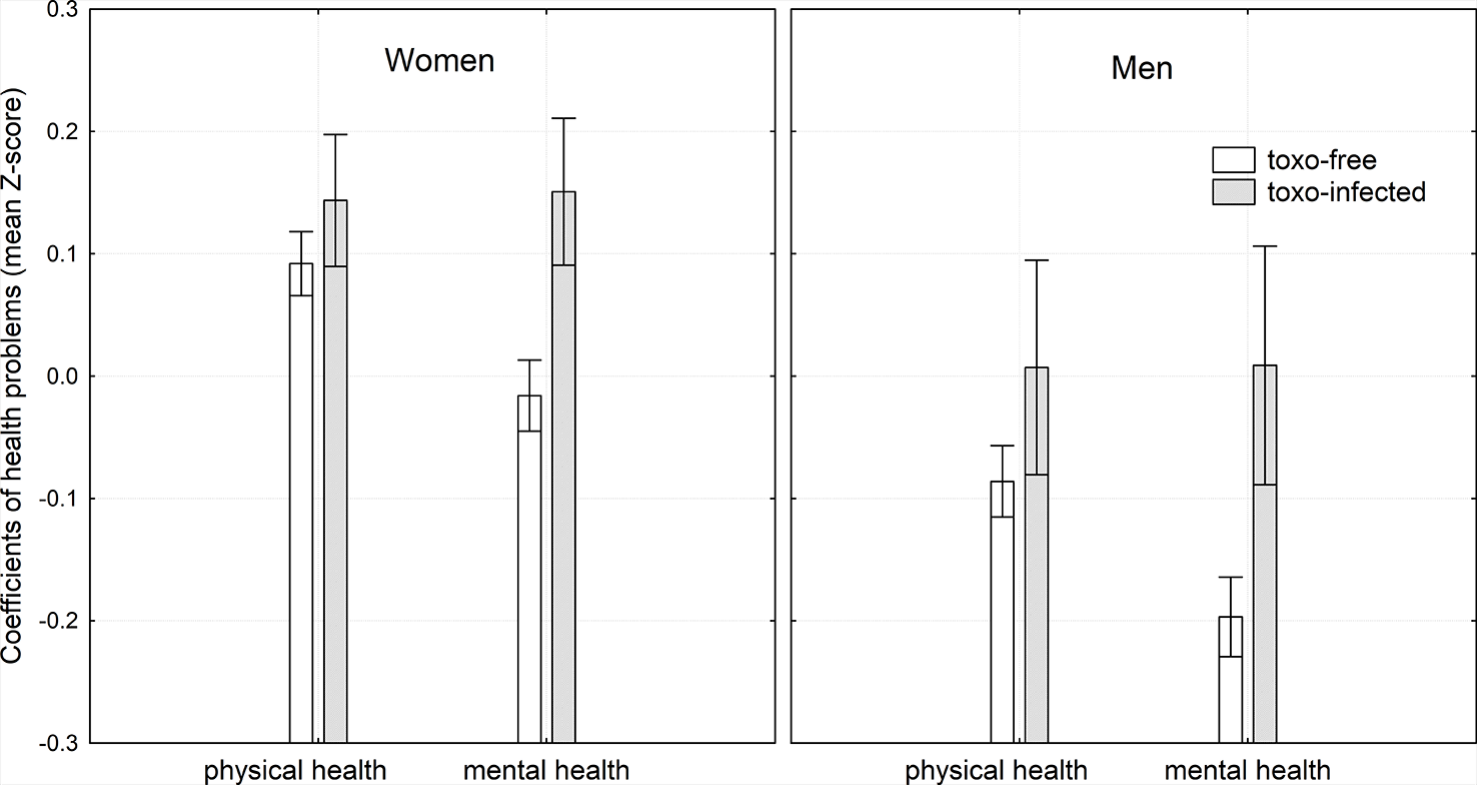
Effects of toxoplasmosis on mental and physical health. The height of the column and the spreads show mean coefficient of health problems and its 95% confidence intervals, respectively. These parameters were computed by general linear model with age and urbanization as covariates, therefore, the figure shows the differences in physical and mental health when these two confounding variables were controlled. For the method of computing the coefficients of physical health problems and mental health problems see Materials and Methods.

**Figure 2:**
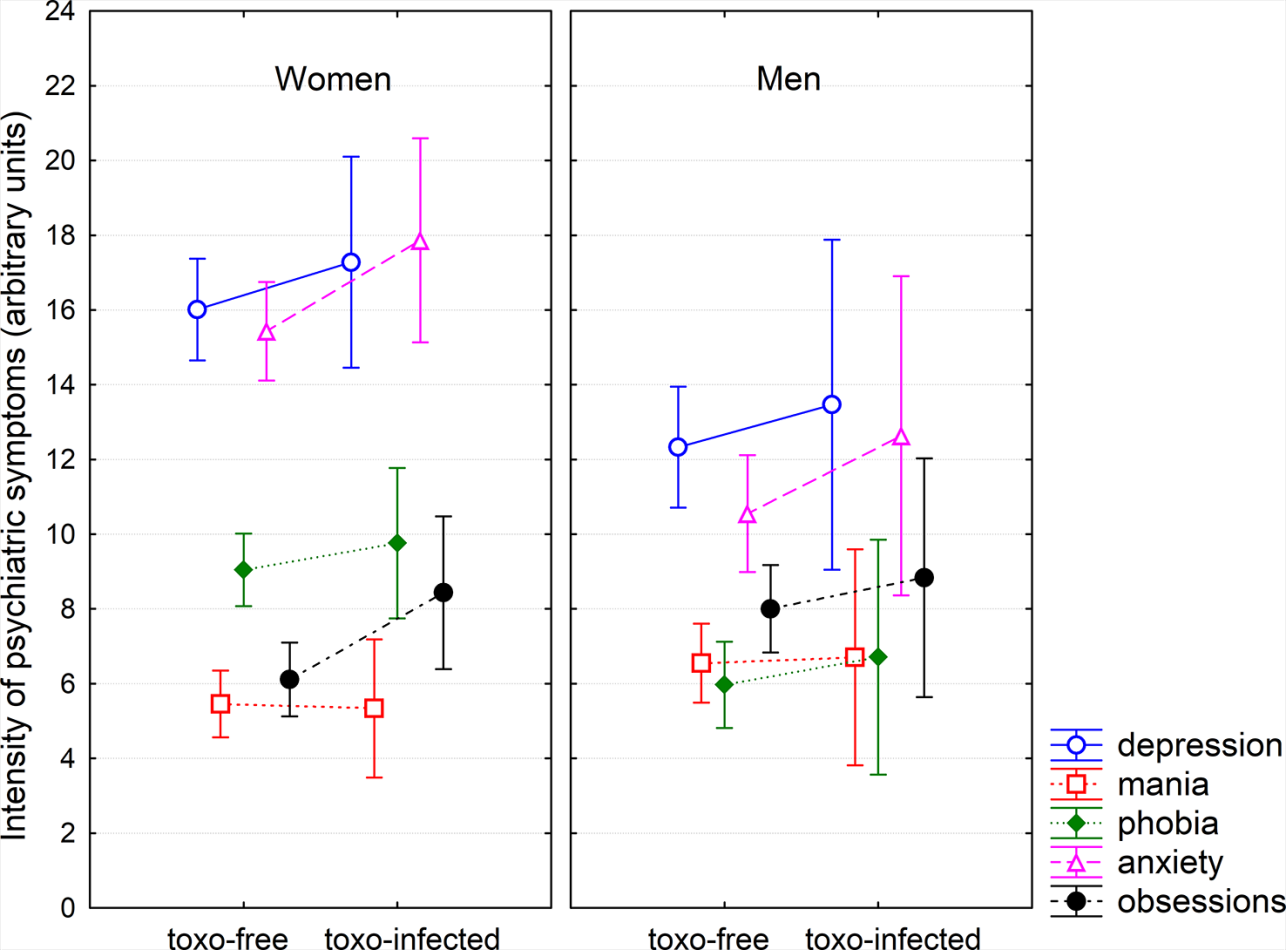
Effects of *Toxoplasma*-seropositivity on reported psychiatric symptoms. The symbols and the spreads show mean intensity of five psychiatric symptoms and its 95% confidence intervals, respectively. These parameters were computed by general linear model with age and urbanization as covariates, therefore, the figure shows the differences in the intensity of psychiatric symptoms when these two confounding variables were controlled.

ANCOVA tests with independent variables of age, urbanization, sex, and toxoplasmosis showed that *Toxoplasma*-seropositive subjects, especially women, reported worse mental and physical health, and more serious symptoms of depression, anxiety, and obsessions, see **Table 1** and **Figures 1** and **2**. To check whether the effect of toxoplasmosis on mental health was mediated by its effect on physical health, we ran the ANCOVA analyses with the variable *physical health problems* included among the covariates. The results were approximately the same as with the model without this covariate — see the last two columns of **Table 1** — suggesting that impaired mental health is not a side-effect of the impaired physical health of the infected subjects.

**Table 1:** Effects of *Toxoplasma*-seropositivity on reported mental health and psychiatric symptoms.

	N	age		urbanization		sex		toxo			toxo-sex		toxo*		
		p	eta^2^	p	eta^2^	p	eta^2^	p	eta^2^	Cohen's d	p	eta^2^	p	eta^2^
coefficient of mental health	6545	**0.000**	0.013	0.137	0.000	**0.000**	0.004	**0.000**	0.006	0.250	0.594	0.000	**0.000**	0.005
coefficient of physical health	6333	**0.000**	0.010	0.708	0.000	**0.000**	0.005	**0.012**	0.001	0.134	0.432	0.000		
depression	3886	**0.000**	0.007	1.000	0.000	**0.000**	0.005	**0.043**	0.001	0.131	0.390	0.000	**0.060**	0.001
mania	2706	**0.000**	0.024	0.804	0.000	**0.149**	0.001	0.604	0.000	0.010	0.759	0.000	0.577	0.000
phobia	2964	**0.000**	0.014	0.242	0.000	**0.000**	0.008	0.347	0.000	0.099	0.585	0.000	0.320	0.000
anxiety	3677	**0.000**	0.015	0.518	0.000	**0.000**	0.009	**0.009**	0.002	0.162	0.714	0.000	**0.015**	0.002
obsessions	2810	**0.000**	0.031	0.348	0.000	0.217	0.001	**0.012**	0.002	0.129	0.624	0.000	**0.000**	0.005

The table shows the results of ANCOVA, i.e., the significance and effect size (eta^2^, which is approximately equal to the fraction of variability of a dependent variable explained by the variability of an independent variable) for mental and physical health and psychiatric symptoms. For the factor toxoplasmosis, the effect size was also shown as the Cohen's d. The last two columns (toxo*) show the result of ANCOVA in which not only age and urbanization, but also physical health, were controlled. The p value < 0.00005 is coded as 0.000; the associations significant after the Benjamini-Hochberg correction for multiple, five or six (the last ANCOVA analysis) tests are printed in bold.

Next, we explored which specific mental and physical health problems were characteristic for the *Toxoplasma*-infected subjects. Here we tested men and women separately using the non-parametric partial Kendall correlation test with age and urbanization as confounding variables. The results showed that toxoplasmosis correlated with all sources of physical and mental health related variables, except the number of visits to primary care doctors and the intensity of suffering from mania. All significant associations except the number of visits of a practical doctor, indicated the worse mental and physical health of the *Toxoplasma*-infected subjects (**Table 2**). To check for the robustness of the results, we separately analyzed a subset of about 800 respondents who were tested for toxoplasmosis in our lab, provided their identification code at the end of the questionnaire, and whose toxoplasmosis status was therefore possible to check rigorously. For this set 2, the associations were non-significant after the correction for multiple tests, however, the strength of the originally significant effects (corresponding partial Kendall Taus) were approximately the same (**Table 2**).

**Table 2:** Association of toxoplasmosis with physical- and mental-health related variables.

	All		Men		Women	
	set 1	set 2	set 1	set2	set 1	set 2
subjective physical health	**-0.026**	-0.012	**-0.034**	-0.028	-0.005	0.026
subjective mental health	**-0.073**	-0.075	**-0.058**	-0.033	**-0.061**	-0.069
drugs prescribed	**0.034**	0.034	-0.011	-0.003	**0.060**	0.050
drugs non-prescribed	**0.040**	0.030	0.015	-0.031	**0.041**	0.053
primary care doctor	-0.022	-0.008	**-0.028**	0.001	**-0.021**	-0.022
antibiotics	**0.003**	-0.008	-0.010	-0.018	-0.004	-0.028
medical specialists	**0.049**	0.058	**0.051**	0.050	**0.025**	0.025
psychiatric problems diagnosed by	**0.063**	0.047	**0.040**	0.002	**0.060**	0.058
problems undiagnosed by doctors	**0.043**	0.009	**0.057**	-0.022	**0.029**	0.015
number of psychiatric diseases	**0.067**	0.073	**0.067**	0.042	**0.054**	0.072
depression	**0.043**	0.018	**0.030**	-0.015	**0.039**	0.037
mania	0.006	0.021	**0.037**	0.031	-0.007	0.028
phobia	**0.021**	0.013	**0.032**	-0.043	0.002	0.002
anxiety	**0.048**	0.028	**0.048**	0.057	**0.032**	-0.002
obsessions	**0.021**	-0.008	**0.039**	-0.013	0.012	0.006
coefficient of physical health	**0.037**	0.051	**0.029**	0.005	**0.022**	0.049
coefficient of mental health	**0.072**	0.057	**0.071**	0.056	**0.055**	0.036

Partial Kendall Taus computed of partial Kendall correlation (age and urbanization controlled) for all subjects who provided the information concerning their toxoplasmosis status (set 1) and for about 800 subjects who signed the questionnaire and whose toxoplasmosis status was checked in our file. The negative Taus correspond to worse health in subjective health problems and to better health in other variables. The associations significant after the Benjamini-Hochberg correction for multiple tests are printed in bold.

The participants were asked to indicate which mental health disorder(s) they suffer from using a checklist with 24 disorders. **Table 3** shows that the *Toxoplasma*-seropositive subjects reported higher prevalences of several mental health disorders, namely autism (OR = 4.78, C.I._95_ = 1.55-14.52), schizophrenia (OR = 3.33, C.I._95_ = 1.32-7.82), attention deficit hyperactivity disorder (OR = 2.50, C.I._95_ = 1.58-3.86), obsessive compulsive disorder (OR = 1.86, C.I._95_ = 1.28-2.66), antisocial personality disorder (OR = 1.63, C.I._95_ = 1.06-2.44), learning disabilities (OR = 1.59, C.I._95_ = 1.08-2.28), and anxiety disorder (OR = 1.48, C.I._95_ = 1.17-1.86).

**Table 3:** Prevalence of common mental health disorders in *Toxoplasma*-seropositive and *Toxoplasma*-seronegative subjects and their association with toxoplasmosis measured with logistic regression.

	Women						Men						Odds Ratio		
	T-D-	T-D+	T-D+	T+D-	T+D+	T+D+	T-D-	T-D+	T-D+	T+D-	T+D+	T+D+	Women	Men	All
Major depression	1715	194	10.2%	415	53	11.3%	1195	82	6.4%	157	11	6.5%	1.14	1.02	1.13
Bipolar disorder	1850	59	3.1%	450	18	3.8%	1238	39	3.1%	166	2	1.2%	1.43	0.38	1.09
Schizophrenia	1903	6	0.3%	464	4	0.9%	1268	9	0.7%	164	4	2.4%	2.82	**3.70**	**3.34**
General anxiety disorder	1629	280	14.7%	375	93	19.9%	1164	113	8.8%	147	21	12.5%	**1.48**	1.44	**1.48**
Alcohol use disorder	1871	38	2.0%	454	14	3.0%	1215	62	4.9%	161	7	4.2%	1.48	0.85	1.17
Gambling	1905	4	0.2%	466	2	0.4%	1263	14	1.1%	166	2	1.2%	1.88	1.01	1.28
Drug use disorder	1892	17	0.9%	467	1	0.2%	1261	16	1.3%	164	4	2.4%	0.25	1.91	0.85
Posttraumatic stress disorder	1808	101	5.3%	444	24	5.1%	1254	23	1.8%	162	6	3.6%	0.97	2.04	1.12
Obsessive compulsive dis.	1841	68	3.6%	442	26	5.6%	1209	68	5.3%	152	16	9.5%	**1.80**	**1.94**	**1.86**
Panic disorder	1806	103	5.4%	439	29	6.2%	1245	32	2.5%	164	4	2.4%	1.19	0.96	1.14
Insomnia primary	1830	79	4.1%	442	26	5.6%	1219	58	4.5%	161	7	4.2%	1.29	0.93	1.21
Learning disabilities	1827	82	4.3%	445	23	4.9%	1219	58	4.5%	152	16	9.5%	1.29	**2.29**	**1.59**
Borderline person. disorder	1878	31	1.6%	461	7	1.5%	1257	20	1.6%	164	4	2.4%	1.03	1.55	1.18
Antisocial person. disorder	1848	61	3.2%	450	18	3.8%	1233	44	3.4%	155	13	7.7%	1.39	**2.26**	**1.63**
Attention deficit hyperactivity dis.	1870	39	2.0%	451	17	3.6%	1247	30	2.3%	155	13	7.7%	**2.00**	**3.50**	**2.50**
Phobias	1551	358	18.8%	386	82	17.5%	1154	123	9.6%	157	11	6.5%	0.97	0.64	0.91
Bulimia, anorexia	1848	61	3.2%	455	13	2.8%	1274	3	0.2%	168	0	0.0%	0.99	0.00	0.94
Burn-out syndrome	1736	173	9.1%	423	45	9.6%	1113	164	12.8%	140	28	16.7%	1.03	1.34	1.14
Sexual disorder	1869	40	2.1%	461	7	1.5%	1228	49	3.8%	160	8	4.8%	0.70	1.28	0.91
Asperger syndrome	1892	17	0.9%	461	7	1.5%	1263	14	1.1%	164	4	2.4%	1.63	2.16	1.87
Autism	1906	3	0.2%	467	1	0.2%	1272	5	0.4%	163	5	3.0%	1.23	**7.83**	**4.78**
Other mental disorder	1824	85	4.5%	442	26	5.6%	1242	35	2.7%	163	5	3.0%	1.26	1.08	1.24

The first twelve columns show the prevalence of disorders in particular subpopulations (T-D- Toxoplasma-free, disorder-free, T-D+ Toxoplasma free, disorder positive, T+D-Toxoplasma-infected, disorder-free, T+D+Toxoplasma-infected, disorder positive) and the last two columns show Odds Ratio and significance (p-level) computed using logistic regression with toxoplasmosis, sex, and age as independent variables. The number of subjects reporting the results of a serological test for toxoplasmosis and providing the information about their mental health disorders was 3,800. The associations significant after the correction for multiple tests are printed in bold.

## Discussion

The main finding of this study is the robust and specific effect of latent toxoplasmosis, or, more precisely, the presence of anamnestic titres of anti-*Toxoplasma* antibodies, on mental health symptoms and disorders. The most characteristic symptom associated with *Toxoplasma*-seropositivity was increased anxiety and the typical toxoplasmosis associated disorders were autism, schizophrenia, attention deficit hyperactivity disorder, obsessive compulsive disorder, antisocial personality disorder, learning disabilities, and anxiety disorder. We observed also a positive trend for Asperger syndrome.

The association of toxoplasmosis with schizophrenia has been confirmed by many studies, for reviews see (3, 4) and a similar association has been documented for obsessive compulsive disorder (15–17) and anxiety disorder (18). The association between toxoplasmosis and autism has been suggested on the basis of three case-control studies and also on various indirect evidence, for review see (1, 19). The lack of association between toxoplasmosis and major (unipolar) depression and panic disorder is in agreement with most of published data (4, 10, 18, 20). As far as we know, the strong association of toxoplasmosis with attention deficit hyperactivity disorder, and the relatively strong association with antisocial personality disorder have not been reported. In contrast, we did not confirm the association of toxoplasmosis with bipolar disorder, which has been reported in about 10 studies, for review and meta-analysis see (4).

The influence of *T. gondii* on the development of psychiatric disorders is most probably mediated both by an immune reaction of the brain to *T. gondii* and by the biochemical activity of the parasite itself. Interferon-gamma secreted in response to toxoplasmosis maintains this infection in a latent form because it induces astrocytes to synthetize indoleamine-2, 3-dioxygenase (IDO), the enzyme responsible for tryptophan degradation *via* the kynurenine metabolic pathway (21, 22). It results in both a lack of tryptophan, an amino acid essential for *T. gondii* replication, and increased levels of the final products of kynurenine pathway. Tryptophan is degraded by IDO into kynurenine which is either metabolized to kynurenic acid, an antagonist of the glutamate NMDA (N -methyl- D -aspartate) receptor or hydroxylated into quinolinate, a potent NMDA neurotoxic agent (23). These metabolites exert both neurotoxic (quinolinate) and pro-psychotic (kynurenic acid) effects and can also influence the neurotransmitter balance (24). However, through the medium of two genes analogical to the human gene for tyrosine hydroxylase, *T. gondii* also directly enhances dopaminergic activity that is critical for the development of schizophrenia, autism, and other mental disorders (3, 4, 17). Hence, dopaminergic and glutamatergic systems (through both agonism and antagonism of NMDA receptors) are affected by *T. gondii* and could represent the mediating factors between toxoplasmosis and mental disorders.

Mental disorders with an identified association with toxoplasmosis in our sample correspond with the role of these pathophysiological pathways. The highest association has been identified for autism spectrum disorders, schizophrenia, and attention deficit hyperactivity which are related to both aberrant neurodevelopment and to the glutamatergic system and dopamine dysregulation (6, 25).

The dopaminergic upregulation mediated by tyrosine hydroxylase activity of *T. gondii* may increase the risk of other disorders of lower ORs like obsessive compulsive disorder (26), antisocial personality disorder (27), and anxiety disorder (28). We also speculate that increased dopaminergic signaling could prevent the increased risk of disorders characterized by a blunted mesolimbic reward mechanism as documented in depression on other mood disorders (29, 30).

## Strength and Limits of the Present Study

Our study probably involves the largest ever population of *Toxoplasma*-tested participants — the usual population size in similar studies is at least one order of magnitude smaller. Our study was exploratory and hypothesis-free in the sense that all main mental health disorders have been analyzed and all results, both positive and negative, have been reported. This approach remediates the well-known problems of the drawer and the cherry picking artifacts — the problems of reporting only positive or “interesting” results of studies.

The most serious limitation of the present study is that the participants (about 0.5% of the inhabitants of Czechia) have been self-selected and therefore they probably do not represent a typical Czech population. The study primarily concerned sexual behavior and sexual preferences and was addressed and promoted as “Human sexuality questionnaire”. Therefore, mainly the subjects who were interested in sexuality-related topics took part in it and finished the whole 110-min questionnaire. The term “evolutionary psychological and parasitological hypotheses” (not *Toxoplasma* or toxoplasmosis) was mentioned only in the information on the first page of the questionnaire and therefore probably played only a marginal role, if any, in the process of auto-selection of the participants of the study. Moreover, the *Toxoplasma*-infected and *Toxoplasma*-free participants had no reason to answer the health-related questions differently (except if they really differed in health). Also, there is no reason to suppose that the association between *Toxoplasma* infection and mental health would differ between the subjects who voluntarily participate in a sex-related study and the general population. In fact, the prevalences of mental health disorders in our internet sample are comparable with those published earlier for Europe (31). For example, the prevalence of alcohol use disorder in Europe was 3.4% (3.2% in our set), schizophrenia 1.2%, (0.6% in our set), unipolar depression 6.9% (8.9% in our set), bipolar disorder 0.9% (3.1% in our set, which better corresponds to other published data), panic disorder 1.8% (4.4% in our set), anxiety disorder 1.7%–3.4% (13.3% in our set — which, again, better corresponds to other data), OCD 0.7% (4.7% in our set, which better corresponds to other published data), posttraumatic stress disorder 1.1%–2.9% (4.0% in our set), anorexia bulimia 1.5% (2.0% in our set), phobias 10.7% (15.0% in our set). Drug use disorder 0.4%–2.2% (1.0% in our set), borderline personality disorder 0.7% (1.6% in our set), antisocial personality disorder 0.6% (3.6% in our set), insomnia 7% (4.5% in our set), attention deficit hyperactivity disorder 3.3% (2.6% in our set).

Another serious limitation of the present study was that the subjects self-reported their mental health status, including the presence of particular mental health disorders, as well as their infection status itself. This is a trade-off for being able to study the interaction between acquired infections and mental health problems on a large enough scale. Our previous analyses of a sample of 3,827 subjects who had been tested in our laboratory for *T. gondii* seropositivity and later registered as our internet volunteers showed that the information concerning toxoplasmosis is mostly (99.5%) correct (32). Still, it is highly probable that a certain fraction of raters misreport their psychiatric diagnoses and especially their self-diagnoses. The results of Monte Carlo modeling, however, showed that stochastic errors caused by misdiagnosing and misreporting health status can result only in false negative results of a study, i.e., the failure to detect existing associations, not false positive results of a study, i.e., detecting non-existing associations (17). Moreover, the current study showed a similar pattern of associations for the main population (set 1) and for the smaller subset (set 2) of subjects with reliable information about their *Toxoplasma* status.

We did not control for the effect of the Rh phenotype in the present study. It has been shown recently, however, that Rh negative women express increased, while Rh-positive women express decreased depression, obsession, and several other facets of neuroticism measured with the N-70 inventory (33). Similarly, the ecological study performed on the set of 65 countries (34) and the cross-sectional study performed on the population of 3,130 people (35) demonstrated worse physical health in Rh negative subjects than in Rh-positive subjects. Our data suggests that the effects of toxoplasmosis on physical and mental health are indeed stronger in the Rh negative than in the Rh positive respondents. However, nearly all effects present in the Rh negative subset, except the strong positive association of toxoplasmosis with major depression that was present only in the Rh negative subjects (OR = 2.3 vs OR = 1.02), were present also in the subset of Rh-positive respondents. It must be stressed, however, that the Rh negative subset contained the health-related data for less than 700 *Toxoplasma*-tested respondents. For reliable analysis we must therefore wait until more data is available.

Given the cross-sectional design of this study, we cannot address the problem of causality. It is not probable that mental health disorders, and especially OCD, could increase the risk of acquiring the *Toxoplasma* infection. However, it is possible that some unknown third factor, such as immunodeficiency, could increase both the risk of mental health disorders and *Toxoplasma* infections. In the light of already published data and our knowledge of the biology of the studied neuropathogen, however, the most parsimonious interpretation of the observed association is the positive effect of the infections on the rate of specific mental health disorders.

The effect sizes of most of the observed associations between toxoplasmosis and quantitative health-related variables (partial Kendall Taus) might seem relatively low. This is, however, a rather universal problem of studies performed in highly genetically polymorphic populations of animals and humans living under natural heterogeneous conditions. Even the factors that have very serious impacts on public health can usually explain only a relatively small part of the variation of the quantitative traits under study. The comparison with other studies showed that the effect sizes of the observed associations (partial Kendall Taus) were similar to those of cigarettes smoking, alcohol and drugs consumption, and high BMI (36). The situation was different with binary variables presence/absence of particular mental health disorders. Here the observed size of the effects, measured as OR, was very high, for example, 4.78 for autism, and 3.33 for schizophrenia.

## Concluding Remarks

Our results obtained in the cross-sectional study performed on a cohort of more than 6,300 subjects tested for toxoplasmosis suggests that the pathogen has strong effects on the rate of several common mental health disorders, including psychoses. The results of analyses of covariance also suggest that the impaired mental health is not a side effect of impaired health in the infected people, as the effects remained intact even when the physical health of respondents were statistically controlled. The results of current study suggest that despite seemingly asymptomatic course of latent toxoplasmosis, *Toxoplasma* could play a privileged role in the etiology of mental disorders.

## Data Availability Statement

The datasets generated for this study are available on request to the corresponding author and are also available at figshare: https://doi.org/10.6084/m9.figshare.9335081.v1.

## Ethics Statement

The studies involving human participants were reviewed and approved by the Ethical Committee of the Faculty of Science, Charles University. Written informed consent for participation was not required for this study in accordance with the national legislation and the institutional requirements.

## Author Contributions

JF designed the study and analyzed the data. Both JF and JH collected the data and wrote the manuscript.

## Conflict of Interest

The authors declare that the research was conducted in the absence of any commercial or financial relationships that could be construed as a potential conflict of interest.

## References

[1] Flegr J Escudero DQ Impaired health status and increased incidence of diseases in *Toxoplasma*-seropositive subjects - an explorative cross-sectional study. Parasitology (2016) 143:1974–89. 10.1017/S0031182016001785 27719690

[2] Flegr J Prandota J Sovickova M Israili ZH Toxoplasmosis - A global threat. Correlation of latent toxoplasmosis with specific disease burden in a set of 88 countries. PLoS One (2014) 9(3):9. 10.1371/journal.pone.0090203 PMC3963851 24662942

[3] Torrey EF Bartko JJ Yolken RH *Toxoplasma gondii* and other risk factors for schizophrenia: an update. Schizophr Bull (2012) 38:642–7. 10.1093/schbul/sbs043 PMC3329973 22446566

[4] Sutterland AL Fond G Kuin A Koeter MW Lutter R Van Gool T Beyond the association. *Toxoplasma gondii* in schizophrenia, bipolar disorder, and addiction: systematic review and meta-analysis. Acta Psychiatr Scand (2015) 132:161–79. 10.1111/acps.12423 25877655

[5] Niebuhr DW Cowan DN Millikan AM Yolken R Li Y Weber N Risk of schizophrenia and antibodies to *Toxoplasma gondii* among U.S. military personnel. Schizophr Bull (2007) 33:243–4.

[6] Horáček J Flegr J Tintera J Verebova K Spaniel F Novak T Latent toxoplasmosis reduces gray matter density in schizophrenia but not in controls: voxel-based-morphometry (VBM) study. World J Biol Psychiatry (2012) 13:501–9. 10.3109/15622975.2011.573809 21599563

[7] Wang HL Wang GH Li QY Shu C Jiang MS Guo Y Prevalence of *Toxoplasma* infection in first-episode schizophrenia and comparison between *Toxoplasma*-seropositive and *Toxoplasma*-seronegative schizophrenia. Acta Psychiatr Scand (2006) 114:40–8. 10.1111/j.1600-0447.2006.00780.x 16774660

[8] Holub D Flegr J Dragomirecka E Rodriguez M Preiss M Novak T Differences in onset of disease and severity of psychopathology between toxoplasmosis-related and toxoplasmosis-unrelated schizophrenia. Acta Psychiatr Scand (2013) 127:227–38. 10.1111/acps.12031 23126494

[9] Celik T Kartalci S Aytas O Akarsu GA Gozukara H Unal S Association between latent toxoplasmosis and clinical course of schizophrenia - continuous course of the disease is characteristic for *Toxoplasma gondii*-infected patients. Folia Parasitol (2015) 62. 10.14411/fp.2015.015 25960559

[10] Flegr J Neurological and neuropsychiatric consequences of chronic *Toxoplasma* infection. Clin Microbiol Rep (2015) 2. 10.1007/s40588-015-0024-0

[11] Flegr J Kuba R The relation of *Toxoplasma* infection and sexual attraction to fear, danger, pain, and submissiveness. Evol Psychol (2016) 14. 10.1177/1474704916659746

[12] Kankova S Flegr J Calda P The influence of latent toxoplasmosis on women's reproductive function: four cross-sectional studies. Folia Parasitol (2015) 62. 10.14411/fp.2015.041 26278510

[13] Flegr J Horáček J Toxoplasmosis, but not borreliosis, is associated with psychiatric disorders and symptoms. Schizophr Res (2018) 197:603–4. 10.1016/j.schres.2018.02.008 29459052

[14] Benjamini Y Hochberg Y Controlling the false discovery rate: A practical and powerful approach to multiple testing. J Roy Stat Soc B Met (1995) 57:289–300. 10.1111/j.2517-6161.1995.tb02031.x

[15] Miman O Mutlu EA Ozcan O Atambay M Karlidag R Unal S Is there any role of *Toxoplasma gondii* in the etiology of obsessive-compulsive disorder? Psychiatry Res (2010) 177:263–5. 10.1016/j.psychres.2009.12.013 20106536

[16] Xiao Y Yin JG Jiang N Xiang M Hao LL Lu HJ Seroepidemiology of human *Toxoplasma gondii* infection in China. BMC Infect Dis (2010) 4:165. 10.1186/1471-2334-10-4 PMC2818656 20055991

[17] Flegr J Horáček J *Toxoplasma*-infected subjects report an obsessive-compulsive disorder diagnosis more often and score higher in obsessive-compulsive inventory. Eur Psychiat (2017a) 40:82–7. 10.1016/j.eurpsy.2016.09.001 27992837

[18] Gale SD Brown BL Berrett A Erickson LD Hedges DW Association between latent toxoplasmosis and major depression, generalised anxiety disorder and panic disorder in human adults. Folia Parasitol (2014) 61:285–92. 10.14411/fp.2014.038 25185399

[19] Prandota J Elleboudy NAF Ismail KA Zaki OK Shehata HH Increased seroprevalence of chronic toxoplasmosis in autistic children: Special reference to the pathophysiology of IFN-gama and NO overproduction. Int J Neurol Res (2015) 1:102–22. 10.17554/j.issn.2313-5611.2015.01.30

[20] Flegr J Hodny Z Cat scratches, not bites, are associated with unipolar depression - cross-sectional study. Parasit (2016) Vectors 9. 10.1186/s13071-015-1290-7 PMC4700762 26728406

[21] Silva NM Rodrigues CV Santoro MM Reis LF Alvarez-Leite JI Gazzinelli RT Expression of indoleamine 2, 3-dioxygenase, tryptophan degradation, and kynurenine formation during *in vivo* infection with *Toxoplasma gondii*: induction by endogenous gamma interferon and requirement of interferon regulatory factor 1. Infect Immun (2002) 70:859–68. 10.1128/IAI.70.2.859-868.2002 PMC127654 11796621

[22] Hunt NH Too LK Khaw LT Guo J Hee L Mitchell AJ The kynurenine pathway and parasitic infections that affect CNS function. Neuropharmacology (2017) 112:389–98. 10.1016/j.neuropharm.2016.02.029 26924710

[23] Elsheikha HM Busselberg D Zhu XQ The known and missing links between *Toxoplasma gondii* and schizophrenia. Metab Brain Dis (2016) 31:749–59. 10.1007/s11011-016-9822-1 27041387

[24] Krause DL Muller N The relationship between Tourette's syndrome and infections. Open Neurol J (2012) 6:124–8. 10.2174/1874205X01206010124 PMC3514747 23230453

[25] Badescu GM Filfan M Sandu RE Surugiu R Ciobanu O Popa-Wagner A Molecular mechanisms underlying neurodevelopmental disorders, ADHD and autism. Rom J Morphol Embryol (2016) 57:361–6. 27516006

[26] Cotrin JC Fontenelle LF Kohlrausch FB Association analyses reveal gender-specific associations of DAT1 40-bp VNTR and -839C/T polymorphisms with obsessive-compulsive disorder and obsessive-compulsive symptoms. Mol Biol Rep (2019) 46:5155–62. 10.1007/s11033-019-04971-9 31325142

[27] Qayyum A Zai CC Hirata Y Tiwari AK Cheema S Nowrouzi B The role of the catechol-o-methyltransferase (COMT) GeneVal158Met in aggressive behavior, a review of genetic studies. Curr Neuropharmacol (2015) 13:802–14. 10.2174/1570159X13666150612225836 PMC4759319 26630958

[28] Zarrindast MR Khakpai F The modulatory role of dopamine in anxiety-like behavior. Arch Iran Med (2015) 18:591–603. 0151809/AIM.009 26317601

[29] Nestler EJ Carlezon WA Jr. The mesolimbic dopamine reward circuit in depression. Biol Psychiatry (2006) 59:1151–9. 10.1016/j.biopsych.2005.09.018 16566899

[30] Grace AA Dysregulation of the dopamine system in the pathophysiology of schizophrenia and depression. Nat Rev Neurosci (2016) 17:524–32. 10.1038/nrn.2016.57 PMC5166560 27256556

[31] Wittchen HU Jacobi F Rehm J Gustavsson A Svensson M Jonsson B The size and burden of mental disorders and other disorders of the brain in Europe 2010. Eur Neuropsychopharmacol (2011) 21:655–79. 10.1016/j.euroneuro.2011.07.018 21896369

[32] Flegr J Predictors of *Toxoplasma gondii* infection in Czech and Slovak populations: the possible role of cat-related injuries and risky sexual behavior in the parasite transmission. Epidemiol Infect (2017) 145:1351–62. 10.1017/S095026881700019X PMC9203307 28183362

[33] Šebánková B Flegr J Physical and mental health status in *Toxoplasma*-infected women before and three years after they learn about their infection: manipulation or side-effects of impaired health? Front Ecol Evol (2017) 5:144. 10.3389/fevo.2017.00144

[34] Flegr J Heterozygote advantage probably maintains Rhesus factor blood group polymorphism: ecological regression study. PLoS One (2016) 11. 10.1371/journal.pone.0147955 PMC4728066 26811928

[35] Flegr J Hoffmann R Dammann M Worse health status and higher incidence of health disorders in Rhesus negative subjects. PLoS One (2015) 10. 10.1371/journal.pone.0141362 PMC4619848 26495842

[36] Flegr J Preiss M Friends with malefit. The effects of keeping dogs and cats, sustaining animal-related injuries and *Toxoplasma* infection on health and quality of life. PLoS One (2019) 14. 10.1371/journal.pone.0221988 PMC6874301 31756184

[37] Flegr J Horáček J Toxoplasmosis, but not borreliosis, is associated with psychiatric disorders: a cross-sectional survey on 46 thousand of subjects. BioRxiv (2017b). 10.1101/231803

